# An educational intervention study on mandatory reporting of intimate partner violence: changes in knowledge and attitudes among healthcare providers

**DOI:** 10.1186/s12909-024-06120-8

**Published:** 2024-10-11

**Authors:** Thea Beate Brevik, Petter Laake, Stål Bjørkly, Kjartan Leer-Salvesen, Solveig Karin Bø Vatnar

**Affiliations:** 1 Faculty of Health Sciences and Social Care, Molde University College, P.O. Box 2110, 6402 Molde, Norway; 2 Oslo Centre for Biostatistics and Epidemiology, Department of Biostatistics, University of Oslo, Oslo, Norway; 3 Department of Social Work, Volda University College, Volda, Norway; 4 Centre for Research and Education in Forensic Psychiatry, Oslo University Hospital, Oslo, Norway

**Keywords:** Mandatory reporting, Intimate partner violence, Domestic violence, Duty to report, Education, Training, Intervention

## Abstract

**Background:**

Intimate partner violence (IPV) is a major public health concern. Healthcare providers can play a crucial role in reporting cases of IPV or suspected IPV injuries to the police or the criminal justice system, commonly referred to as mandatory reporting. However, mandatory reporting of intimate partner violence (MR-IPV) is a debated topic that can pose complex challenges for healthcare providers. This underscores the importance of training programs to ensure that healthcare providers can fulfill their MR-IPV obligations.

**Methods:**

We developed an educational intervention on MR-IPV and assessed its impact on healthcare providers’ knowledge and attitudes. The study used a pre- and post-test design with three measurement points: baseline (T0), immediately after the intervention (T1), and six months later (T2). The intervention was conducted at a university college in Norway, with data collected between October 2022 and May 2023. The intervention was delivered to 37 healthcare providers who were currently part-time students in mental healthcare. Changes in knowledge and attitudes between T0 and T1, and T0 and T2 were analyzed through nonparametric tests on related samples using the Marginal homogeneity (Stuart–Maxwell) test. Risk differences (RD), along with their corresponding 95% confidence intervals (CI), were calculated for selected categories.

**Results:**

The number of participants knowing the MR law increased from 2.9% at baseline to 62.9% at T1 (RD = 0.60, 95% CI: 0.41—0.79) and to 31.4% at T2 (RD = 0.29, 95% CI: 0.11–0.46). The number of participants reporting knowing relevant criteria increased from 0.0% at baseline to 68.6% at T1 (RD = 0.69, 95% CI: 0.50–0.87) and to 34.3% at T2 (RD = 0.34, 95% CI: 0.16—0.53). We observed several persistent changes in healthcare providers' attitudes towards MR, including finding MR to be a useful instrument and generally complying with MR requirements.

**Conclusions:**

The findings suggest that this educational intervention can have a positive impact on healthcare providers' attitudes and compliance with MR-IPV. Before the intervention, few participants reported knowing the MR law and its application criteria, demonstrating the importance of continuous learning and evidence-based training programs.

**Supplementary Information:**

The online version contains supplementary material available at 10.1186/s12909-024-06120-8.

## Background

Intimate partner violence (IPV) is a major public health concern and includes behavior causing physical, sexual, and psychological harm by a current or former intimate partner [1]. One in three women is expected to experience physical or sexual IPV, or both, during their lifetime [2], underscoring the urgent need for effective prevention. An essential part of a preventive strategy is to ensure that healthcare providers have specialized knowledge to recognize and address IPV adequately. Healthcare providers have a vital role in detecting signs of IPV, providing care, referring victims to support services, and being trusted with the victims’ personal experiences [1–7]. Effective responses can include reporting violence, and healthcare providers can play a crucial role in reporting cases of IPV or suspected IPV injuries to the police or the criminal justice system [8–12]. Many countries and states have adopted mandatory reporting laws concerning IPV, although they vary significantly across countries and states [8–11].

However, mandatory reporting of intimate partner violence (MR-IPV) is a debated topic that that can pose complex challenges for healthcare providers. On one hand, they may be legally responsible for acting on information or suspicion about IPV. Additionally, they can be considered ethically responsible for taking actions, as research has shown that women who have disclosed IPV to healthcare providers can expect them to "do more than just listen" [7]. On the other hand, healthcare providers have reported a lack of knowledge and specific training, which can leave them feeling uncertain and unprepared in clinical practice [6, 13]. Additionally, they have expressed concerns about the time and resources required, the possibility that women may be discouraged from disclosing information, potential breaches of confidentiality and autonomy, the risk of retaliation, and the consequences of unsuccessful prosecutions [8]. This can lead to underreporting, as previous research has indicated that few professionals report IPV cases under MR [9]. These dilemmas underscore the complexity of MR-IPV and the importance of training interventions that address both reporting and legal requirements, as well as response actions, to ensure healthcare providers can fulfill their MR-IPV obligations.

Despite healthcare providers' need for specialized knowledge and training, significant gaps have been identified in current training curricula on IPV for healthcare providers [6, 7]. For training in MR-IPV in particular, a recent systematic review identified only two intervention studies on the topic [9]. The first intervention study was conducted by Allert with colleagues (1997) [14] to improve the identification, treatment, and referral of domestic violence victims by prehospital care providers and emergency department personnel (*N* = 213). The methods covered pre- and post-tests, including a follow-up post-survey three months later, to assess knowledge, attitudes, and procedures related to identifying, treating, and reporting domestic abuse. The study results indicated that, prior to the intervention, 27% of participants were unaware of the MR-IPV law, while after the intervention, 15% of participants were still unable to correctly identify injuries that should be reported under the law. Additionally, prior to the intervention, only 40% of participants knew where to send a report, whereas after the intervention, 71% had learned that injuries should be reported to law enforcement. Interestingly, of the participants who reported providing care to a domestic violence victim during the post-test, only 23% had actually reported the crime. The second intervention study was conducted in 2007 by Aved with colleagues (2007) [15] to challenge dentistry to recognize and respond to family violence (*N* = 1213). Despite only utilizing a survey and post-tests at the end of the training session, the researchers claimed that the training resulted in a systematic change in how dental professionals perceived their role and responsibility in relation to violence and neglect among their patients. The participants generally agreed that educational programs about MR-IPV was important; this included knowing how and where to report IPV (with a mean response of 3.05 on a 4-point scale, where 4 = strongly agree), knowing how to recognize signs and symptoms of abuse (mean 3.05), and knowing the legal responsibilities of reporting (mean 3.08).

### Aim of the study

Given the scarcity of research on educational programs for healthcare providers on MR-IPV, an educational intervention about MR-IPV was deemed the most appropriate method for this study. The study aimed to answer the following research questions:

- Does this educational intervention enhance the healthcare providers’ knowledge regarding MR-IPV?
- If knowledge is increased, does it remain elevated after six months?
- Does the intervention lead to changes in healthcare providers’ attitudes toward MR-IPV?
- If attitudes are changed, do these attitude changes persist after six months?

## Methods

This study used a pre- and post- test design to examine the impact of an educational intervention about mandatory reporting of intimate partner violence (MR-IPV) on the knowledge and attitudes of nurses, social educators, child welfare and social workers – hereinafter called *healthcare providers*.

This study is a part of the Manreport-IPV project, which is a cross-professional project that explores the experiences, awareness, and attitudes regarding MR-IPV among IPV help-seekers and professionals. The main objective of the project is to expand empirical knowledge about MR-IPV, to inform effective prevention strategies against severe IPV and homicides. The project was approved by Oslo University Hospital Data Protection Official (reference number: 22/00221). The Regional Committees for Medical Research Ethics considered the study to be health service research and thereby not within their mandate (reference number: 257644).

### Study setting

This study was conducted in a small city on the west coast of Norway with approximately 32,000 inhabitants. The intervention was carried out at a university college with around 3,500 students. Data for the intervention study was collected between October 2022 and May 2023. In terms of the legal setting, mandatory reporting in Norway is governed by Sect. 196 of the Norwegian Penal Code, which aims to avert ongoing or potential criminal acts and their consequences [16]. The section applies to all adult individuals in Norway, including healthcare providers. Section 196 refers to criminal acts that require reporting, which can involve notifying the police or averting the criminal act or its consequences "by other means". The duty applies when there is information indicating it is "certain or most likely" that the crime will occur. Failing to report in accordance with Sect. 196 is a punishable offense, unless reporting would put the person making the report, their immediate family, or an innocent individual at risk of charges, indictment, or harm to life, health, or well-being [16]. The threshold for invoking this duty is *severe* or *persistent* IPV. Notably, mandatory reporting is not constrained by any professional confidentiality laws [16].

### The educational intervention

We developed an educational intervention, covering client confidentiality and mandatory reporting in general and in the context of IPV. The first session focused on the principles of client confidentiality, highlighting its application and exceptions in cases of harm to oneself or others. The lecture emphasized the need for healthcare workers to be familiar with relevant laws, regulations, and ethical guidelines in their respective fields. The second session underscored the MR law, explaining that it in Norway applies to all individuals, including those bound by confidentiality obligations [16]. Reporting may include notifying the police or other actions to avert harm, and patient consent is not necessary in situations including severe or persistent IPV. The third session focused on IPV, discussing prevalence in Norway and internationally, followed by an overview of barriers to report faced by professionals, such as confidentiality, fear of being wrong, fear of consequences, personal experiences, and negative impressions of health services or other agencies. The fourth session focused on MR-IPV, where the students learned about the law (Sect. 196 pointing to Sect. 282 and 283) and possible measures that can be taken in clinical practice. The final session provided a clinical case scenario about a woman with foreign citizenship visiting an emergency room with facial injuries, and who initially reported IPV as source of the injuries, but later claimed that the injury was accidental. The students individually assessed the case scenario before discussing their assessment in groups and in plenary.

**Fig. 1:**
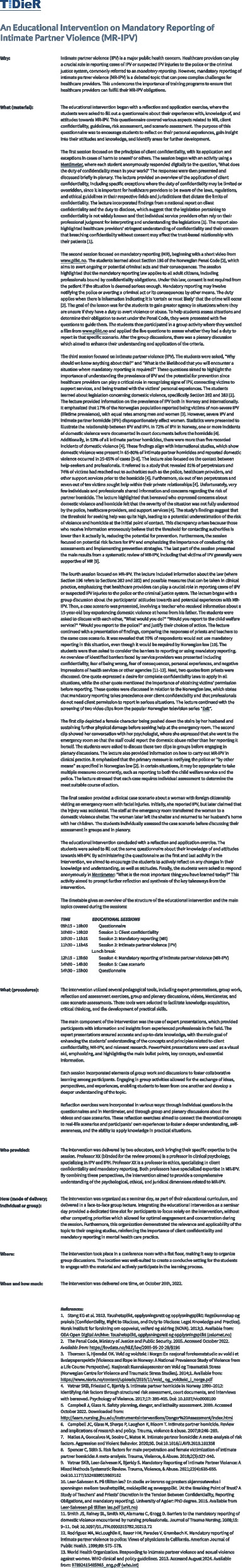
The TIDieR checklist for the educational intervention on mandatory reporting of intimate partner violence (MR-IPV). Pdf uploaded as a separate file

A range of pedagogical tools were carefully selected to create an engaging and interactive learning environment. The tools included expert presentations, group work, reflection and assessment exercises, group and plenary discussions, videos, and case scenario assessments. As part of the reflection and applications exercise, the students were asked to fill out a questionnaire about their experiences with, knowledge of, and attitudes towards MR-IPV. For further details on the intervention's structure and content, please see Fig. 1.

### The questionnaire measurements

The study used a validated questionnaire provided by The Norwegian Institute for Studies of the Medical Profession [17], with specific sections adjusted for the scope of this project. The original covers Likert-scale statements on attitudes, awareness, and experiences on societal and organizational conditions for professional work, ethics, values, and evaluating prioritization in the work context [18–21]. The questionnaire used in the current study encompassed the following sections:

A) Occupational status, working conditions, and working environment
B) Professional experience with IPV
C) Professional experience with MR-IPV
D) Knowledge and attitudes towards mandatory reporting and client confidentiality
E) Knowledge and attitudes towards guidelines and risk assessment
F) Knowledge and attitudes towards a clinical case scenario.

The questions relevant to the present article have been translated into English and are available in Supplementary file 1.

Participants’ professional experiences with IPV were measured according to the Norwegian translation [22] of the revised Conflict Tactics Scale (CTS2) [23]. The questionnaire has demonstrated good validity and reliability and is among the most widely used measures of IPV [24, 25]. *Severe IPV* was defined according to the Norwegian Penal Code for domestic violence [16] and the Norwegian translation of the CTS2 [22, 23]. The definitions were described in the questionnaire before the relevant questions as behavior that caused or could cause serious injury or death; was persistent abuse; and/or involved use of a knife or other dangerous tool/weapon.

The participants were asked about the number of professional encounters they had with individuals who have been subjected to (victims) or have subjected others (perpetrators) to IPV. Their encounters were measured both throughout their careers and within the preceding 12 months. Encounters with victims and perpetrators were measured separately, in line with the majority of IPV research, which treats victims and perpetrators as dichotomized roles. However, there is evidence indicating that bidirectional IPV is the most prevalent form of IPV, with psychological violence being the most commonly reported type of bidirectional violence (e.g., [26, 27]). Acknowledging this, the current study permitted healthcare providers to determine which encounters they considered to fall into the categories of victims and perpetrators.

### Pilot of the intervention study

A pilot study was conducted on March 8, 2021, to test the intervention, with a follow-up after two months on May 10th. Of the 44 participants invited to participate, 33 completed the questionnaire at least once. Their feedback was used to make necessary adjustments for the actual intervention. The response rate in the pilot study decreased from 76% at baseline to 60% after the intervention, which led us to offer a 20 Euro gift card incentive for completing the questionnaire at the two-month follow-up, which resulted in an improved response rate. Based on this positive outcome, we decided to also offer a 20 Euro gift card incentive in the actual intervention study. Additionally, we found that paper questionnaires were completed at a higher rate than the digital version, and we received feedback that several participants experienced technical difficulties with the digital form. Therefore, we made the decision to only provide a paper version on the day of the actual intervention. Finally, participants indicated that completing demographic variables and working experiences two times on the day of the intervention and again two months later was unnecessary and time-consuming. Based on this feedback, we decided to only include Sections A, B, and C, which measure demographic variables and working experiences, at baseline. Sections D, E, and F, which measure knowledge and attitudes, were included in all three measurements to provide a shorter questionnaire, and reduce the time required to complete it.

### Recruitment procedure

**Fig. 2:**
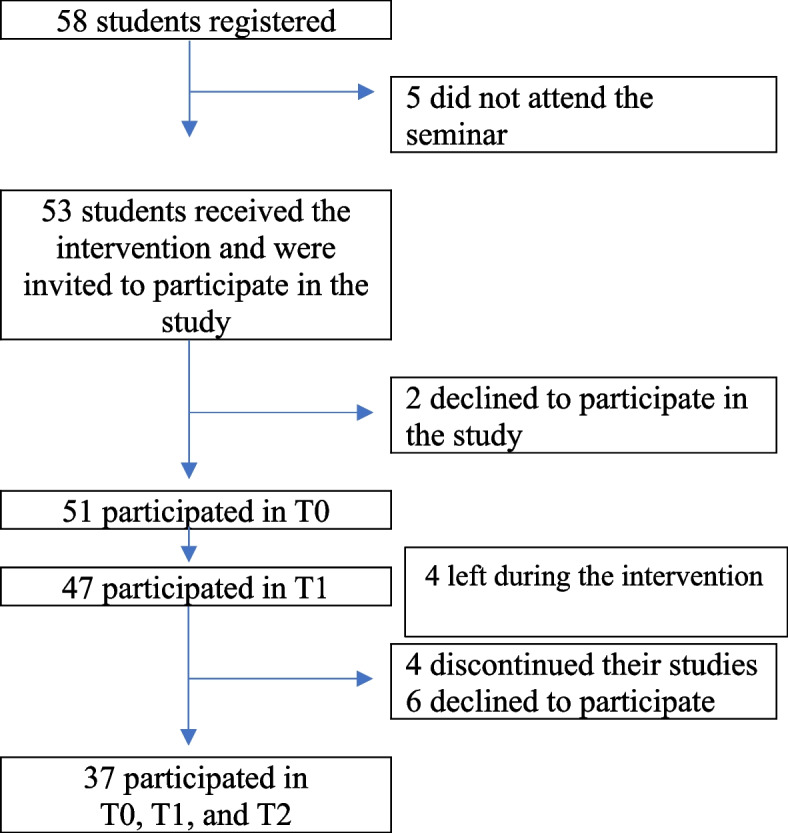
Flow chart of study participants

Invitations to participate in this study were extended to healthcare providers who were currently part-time students at a university college, taking further education in mental healthcare. These participants were selected to ensure that the participants had a fundamental understanding of healthcare principles and ethics and experiences with working as healthcare providers. By building upon their existing knowledge and skills, the intervention could delve deeper into the specific aspects of MR-IPV. The educational intervention was organized as a seminar day, as part of their educational curriculum. The intervention took place on October 20th, 2022. A total of 53 students attended the seminar and received the intervention. As a part of the pedagogical tools that were used to engage learning, all students were asked to complete a questionnaire about their experiences with, knowledge of, and attitudes towards MR-IPV, both in the morning before the education started and immediately after the educational intervention was finished. At the end of the seminar day, all students were invited to take part in the study by providing us with their completed questionnaires. Participation in the study was voluntary, and students were assured of the anonymity and confidential handling of their information. Detailed information about the study, including its purpose, procedures, and voluntary nature, was provided to the students both verbally and in writing. Participants were also informed that choosing not to participate in the study or declining to answer specific questions would not have any consequences for their future work or education. Written informed consent was obtained from all participants. Six months after the intervention, on May 10^th^, 2023, all students were invited to complete the questionnaire a third time. Figure 2 outlines the flow chart of study participants. In total, 37 participants took part in all three measurements, and analysis of these data is the scope of this article.

### Sample

The sample comprised 36 women and one man, aged between 23 and 52 years (mean age 36; SD 8.18). In terms of occupation, there were 21 nurses, 9 social educators, and 7 child welfare and social workers. The average years of working experience were 8.5 years, ranging from 1 to 22 years (SD 6.44). Most participants had encountered individuals who had been subjected to (victims) and/or had subjected others (perpetrators) to physical, psychological, and/or sexual IPV. A total of 68% of the participants had encountered victims during their careers, and 49% had encountered victims in the preceding 12 months. Additionally, 57% of the participants had encountered perpetrators during their careers, whereas 35% had encountered perpetrators in the preceding 12 months. The participants had a diverse range of professional experiences, meeting from 1 to 60 victims and from 1 to 100 perpetrators throughout their careers. Many participants had experiences with *severe* cases of IPV; 54% reported meeting victims of severe IPV (ranging from 1–30 victims), and 46% had met perpetrators of severe IPV (ranging from 1–40 perpetrators). Of the 25 participants reporting encountering victims and/or perpetrators of IPV during their career, only ten participants had ever experienced cases where MR had been required.

### Statistical analyses

It was observed that some participants provided two responses to single questions. To account for this, an imputation process was applied, where the imputed values were randomly selected as a number from the Bernoulli distribution, based on the probability distribution of the item specific responses. Descriptive analyses and imputations were performed in SPSS. Descriptive analyses were performed for all demographic variables from sections A, B, and C of the questionnaire. Items measuring knowledge and attitudes (sections D and E) were analyzed at three time points: baseline (T0), immediately after the intervention (T1), and six months later (T2). Changes in knowledge and attitudes between T0 and T1, and between T0 and T2 were analyzed through nonparametric tests on related samples using the Marginal homogeneity (Stuart–Maxwell) test [28]. A p-value of ≤ 0.05 was considered statistically significant. To analyze changes in the participants’ responses, cross tables were applied to calculate risk differences (RD) with their corresponding 95% confidence intervals (95% CI) for selected categories [29]. Marginal homogeneity and RDs were calculated in Stata 17.

## Results

### Changes in knowledge of MR

At baseline, few participants reported knowing the law of MR in general (2.9%) and within their respective fields (11.8%). Most of the participants reported knowing the MR law to some extent (85.7% in general and 76.5% within their field). In total, 40% of the participants were not informed about the criteria that should be used to make a decision regarding the application of MR within their field, while 60% were informed to some extent. Only 21.6% of the participants were well-informed about the current guidelines within their field. About half of the participants answered that several questions regarding MR were not relevant to them.

The educational intervention significantly increased the participants’ knowledge of MR laws (see Table 1, Chi-square = 22.05, *p* < 0.001 between T0 and T1, and Chi-square = 12.31, *p* = 0.002 between T0 and T2). The number of participants knowing the MR law increased from 2.9% at baseline to 62.9% immediately after the intervention (RD = 0.60, 95% CI: 0.41—0.79) and to 31.4% six months later (RD = 0.29, 95% CI: 0.11–0.46). Similarly, knowledge of MR laws within their respective field increased from 11.8% at T0 to 58.8% at T1 (RD = 0.47, 95% CI: 0.27—0.67) and to 35.3% at T2, although this specific change was not statistically significant at the six-month follow-up.

**Table 1:** Changes in knowledge of and attitudes towards mandatory reporting of intimate partner violence (MR-IPV) between baseline (T0), immediately after the intervention (T1), and six months after the intervention (T2)

**Item**	**T0-T1**			**T0-T2**		
	*Chi-square*	*df*	*p-value*	*Chi-square*	*df*	*p-value*
Do you know the mandatory reporting law?	22.05	2	** < 0.001**	12.31	2	**0.002**
Do you know the mandatory reporting law within your field?	18.29	2	** < 0.001**	8.17	2	0.167
Severe intimate partner violence should always be reported to the police	8.00	2	**0.018**	6.43	2	**0.040**
I am the one who decides whether I will use mandatory reporting	16.76	3	**0.001**	7.41	3	0.060
Client confidentiality takes precedence over mandatory reporting	13.52	3	**0.004**	7.45	3	0.059
Mandatory reporting takes precedence over client confidentiality	9.00	3	**0.030**	6.67	3	0.083
Do you believe the mandatory reporting law considers the central factors in the risk situation	17.44	3	** < 0.001**	14.55	4	**0.006**
Has knowledge of mandatory reporting changed the way you work?	12.79	4	**0.012**	8.33	4	0.080
Do you believe mandatory reporting is a useful tool?	18.22	3	** < 0.001**	10.95	3	**0.012**
Everyone has the right to a physician/therapist/healthcare provider/contact person with absolute client confidentiality	22.30	3	** < 0.001**	4.72	3	0.193
If I disclose confidential information, patients/clients/consumers will lose trust in me, regardless of justification	18.95	4	**0.001**	6.31	4	0.177
If I disclose confidential information, I will feel unprofessional, regardless of justification	18.27	4	**0.001**	10.81	3	**0.013**
The patient's wish matters most; if they don't want to report to the police, I won't do it	19.87	3	** < 0.001**	13.24	3	**0.004**
I find other ways to cooperate with, for example, the police, without using mandatory reporting	11.89	4	**0.018**	5.43	3	0.143
Unless the patient poses a significant risk to others, the help services should not overrule the wishes and decisions of adults	13.70	4	**0.008**	8.28	4	0.082
Mandatory reporting is important to ensure that victims of intimate partner violence do not have to report the risk of severe abuse and/or of intimate partner homicide themselves	4.31	4	0.366	11.40	4	**0.022**
I generally comply with the mandatory reporting requirements when treating patients/clients/consumers	8.53	2	**0.014**	12.97	2	**0.002**
The distinction between the Health Personnel Act and mandatory reporting is unclear	15.58	4	**0.004**	14.30	4	**0.006**
I find that my professional autonomy is reduced due to mandatory reporting	14.44	4	**0.006**	13.54	4	**0.009**
Are you informed about the criteria that should be used to make a decision regarding the application of mandatory reporting within your field?	26.35	2	** < 0.001**	16.11	2	** < 0.001**
I am well-informed about the current guidelines in my field	10.57	4	**0.032**	14.57	4	**0.006**
I often disagree with the recommendations in the guidelines	8.90	3	**0.031**	7.65	3	**0.054**
The guidelines are integrated into the way I work	8.73	3	**0.033**	5.94	3	0.116
Guidelines are often not easily accessible	5.00	4	0.287	10.10	4	**0.039**
I miss a comprehensive record of guidelines	9.70	4	**0.046**	7.42	4	0.116
I have confidence in the guidelines published by my professional union	6.16	3	0.104	7.81	3	**0.050**

The intervention significantly increased the participants’ knowledge of the current guidelines within their field (Chi-square = 10.57, *p* = 0.032 between T0 and T1, and Chi-square = 14.57, *p* = 0.006 between T0 and T2). The intervention also increased their knowledge of the criteria relevant for MR-IPV assessments (Chi-square = 26.35, *p* < 0.001 between T0 and T1, and Chi-square = 16.11, *p* < 0.001 between T0 and T2). The number of participants reporting being informed about the criteria increased from 0.0% at baseline to 68.6% at T1 (RD = 0.69, 95% CI: 0.50–0.87) and to 34.3% at T2 (RD = 0.34, 95% CI: 0.16—0.53).

### Changes in knowledge of and attitudes towards MR-IPV

We observed several changes in knowledge and attitudes related to client confidentiality and MR, especially at T1. Immediately after the intervention, there were significant changes in knowledge regarding whether client confidentiality takes precedence over mandatory reporting, or vice versa. In response to the statement “Client confidentiality takes precedence over mandatory reporting”, significantly more participants answered *farthest from my perception* after the intervention, increasing from 51.4% at T0 to 88.6% at T1 (RD = 0.37, 95% CI: 0.16—0.58). Similarly, significantly more participants answered *closest to my perception* of the statement “Mandatory reporting takes precedence over client confidentiality”, increasing from 72.7% at T0 to 93.9% at T1 (RD = 0.21, 95% CI: 0.02–0.41). Moreover, significantly more participants disagreed with the statement that if they disclose confidential information, patients will lose trust in them, regardless of justification (Chi-square 18.95, *p* = 0.001 between T0 and T1). The intervention also changed the participants' attitudes towards the statement that everyone has the right to a healthcare provider with *absolute* client confidentiality (Chi-square = 22.30, *p* < 0.001 between T0 and T1). Initially, only 17.1% completely disagreed with this statement, but this increased to 62.9% at T1 (RD = 0.46, 95% CI: 0.26—0.65).

We observed several persistent changes in the participants’ attitudes towards MR-IPV that remained significant six months after the intervention. The intervention significantly influenced the participants’ attitudes towards whether MR law considers the central factors in the risk situation (Chi-square = 17.44, *p* < 0.001 between T0 and T1, and Chi-square = 14.55, *p* = 0.006 between T0 and T2). Initially, only 12.9% completely agreed with the statement, but this increased to 67.7% at T1 (RD = 0.55, 95% CI: 0.32—0.78) and to 50.0% at T2 (RD = 0.38, 95% CI: 0.14—0.61). The intervention also had a significant impact on the participants’ attitudes towards the usefulness of MR as an instrument (Chi-square = 18.22, *p* < 0.001 between T0 and T1, and Chi-square = 10.95, *p* = 0.012 between T0 and T2). Before the intervention, 34.3% completely agreed with this statement, and this increased to 85.7% at T1 (RD = 0.51, 95% CI: 0.32—0.71) and to 71.4% at T2 (RD = 0.37, 95% CI: 0.15—0.60). Furthermore, all participants (100%) believed at T1 that severe IPV should always be reported to the police (RD = 0.22, 95% CI: 0.06—0.38), and this was maintained by 94.6% of participants six months later (RD = 0.16, 95% CI: 0.02—0.31).

### Changes in attitudes towards MR

Several persistent changes in the participants’ attitudes related to barriers to using MR in clinical practice were observed. The intervention significantly changed the participants’ attitudes towards the statement “If I disclose confidential information, I will feel unprofessional, regardless of justification” (Chi-square = 18.27, *p* = 0.001 between T0 and T1, and Chi-square = 10.81, *p* = 0.013 between T0 and T2). Initially, only 24.3% of the participants completely disagreed with this statement, but this increased to 51.4% at T1 (RD = 0.27, 95% CI: 0.10—0.44). A similar finding was observed with the statement “The patient's wish matters most; if they don't want to report to the police, I won't do it” (Chi-square = 19.87, *p* < 0.001 between T0 and T1, and Chi-square = 13.24, *p* = 0.004 between T0 and T2). Initially, 24.3% of the participants completely disagreed with this statement, but this increased to 67.6% at T1 (RD = 0.43, 95% CI: 0.25—0.62).

Notably, some findings indicate that the intervention did not result in immediate changes in certain attitudes; however, a significant change in attitudes was observed six months later. For instance, participants were asked whether “guidelines are often not easily accessible”. At baseline, 43.3% agreed (totally or partially) and 32.4% disagreed (totally or partially) with the statement. There was no significant change in responses immediately after the intervention, which was expected since it was administered on the same day. However, a significant change was observed six months later (Chi-square = 10.10, *p* = 0.039), with 56.8% disagreeing (totally or partially, RD = 0.24, 95% CI: 0.01—0.47), indicating that an increased focus on MR-IPV may have made more participants aware of the guidelines existing in their clinical work. Similar results were obtained for the statement that MR is important to ensure that victims of IPV do not have to report the risk of severe abuse and/or intimate partner homicide themselves, with no statistical changes noted immediately post-intervention but a significant change observed six months later (Chi-square = 11.40, *p* = 0.002), where 91.9% of the participants responded at T2 that they completely agreed the statement.

Interestingly, significant reductions were observed in the proportion of participants who responded "not relevant to me" for several items. For instance, in response to the statement "I find that my professional autonomy is reduced due to mandatory reporting", there was a decrease in participants responding "not relevant to me", from 45.9% at T0 to 10.8% at T1 (RD = -0.35, 95% CI: -0.55, -0.15) and to 13.5% at T2 (RD = -0.32, 95% CI: -0.50, -0.15). At the same time, the number of participants disagreeing with this statement increased from 37.8% at T0 to 75.7% at T1 (RD = 0.35, 95% CI: 0.14—0.57) and to 54.1% at T2 (RD = 0.30, 95% CI: 0.11—0.49). Similar findings were observed regarding the statement that the distinction between the Health Personnel Act and mandatory reporting is unclear. These findings suggest that the educational intervention had a substantial impact on how participants perceived the relevance of the topic.

Finally, the intervention significantly increased the number of participants stating that they generally comply with MR requirements (Chi-square = 8.53, *p* = 0.014 between T0 and T1, and Chi-square = 12.97, *p* = 0.002 between T0 and T2). Initially, 27.8% completely agreed with this statement, and this increased to 50.0% at T1 (RD = 0.22, 95% CI: 0.02—0.42). Six months later, it had increased to 59.5% (RD = 0.32, 95% CI: 0.11—0.54).

## Discussions

### Main findings

Healthcare providers need specialized knowledge to fulfill their obligations of MR-IPV. However, despite the laws surrounding IPV having changed in many countries and the World Health Organization’s and the United Nations’ focus on preventing IPV, there is a significant gap in current training curricula on IPV and MR for healthcare providers. In this study, we developed an educational intervention on MR-IPV, which had a positive impact on the healthcare providers' knowledge and several attitudes. This finding concurs with previous intervention studies on the same topic [14, 15].

Our main findings were that the intervention significantly increased healthcare providers' knowledge about MR laws, guidelines, and criteria relevant to assessing whether MR-IPV applies in clinical contexts. This knowledge remained elevated six months later, although we note that healthcare providers exhibited reduced certainty at this point. We observed several persistent changes in healthcare providers' attitudes towards MR, including finding MR to be a useful instrument and generally complying with MR requirements. The intervention also had an immediate impact on healthcare providers' attitudes related to client confidentiality and MR. After the intervention, most healthcare providers correctly responded that mandatory reporting takes precedence over client confidentiality, and many disagreed with the statement that everyone has the right to a healthcare provider with *absolute* client confidentiality. One observed trend was the larger changes in knowledge and attitudes immediately following the intervention compared to six months later, indicating a more immediate impact. This finding highlights the need for continued training. Lastly, the intervention had a substantial impact on healthcare providers' perception of the relevance of the topic, with significant reductions in the proportion of participants who responded "not relevant to me" for several items.

### Changes in knowledge of and attitudes towards MR-IPV

Previous research has indicated that professionals may have low compliance with MR requirements for reporting IPV [9]. Client confidentiality and fear of breaking patients' trust are among the barriers that can hinder healthcare providers from reporting [30, 31]. Rodriguez and colleagues [31] found that 59% of physicians would not comply with MR-IPV if the patient objected. Effective training interventions should address both barriers and response strategies [6], as well as reporting and legal obligations [15]. The educational intervention developed for this study covered several topics related to MR-IPV, including a strong emphasis on the legal context in Norway and barriers to reporting. Healthcare providers engaged in various didactic activities and reflection exercises that focused on understanding and applying the criteria for identifying MR-IPV. The goal was to empower healthcare providers and enhance their ability to navigate situations where they were unsure whether they have a duty to avert violence or abuse. Previous studies including pre- and posttests of professionals participating in training courses have indicated changes in awareness [14, 15], but no changes in practicing MR-IPV were measured. Even if we have no external measures of changes in practice, the intervention led to significant changes in healthcare providers' self-reported compliance with MR-IPV. Moreover, significantly more healthcare providers indicated that MR-IPV would not reduce their professional autonomy and that they were willing to report IPV against patients' wishes, in accordance with the Norwegian MR-IPV law. These findings suggest that educational interventions can have an impact on healthcare providers' attitudes and compliance with MR-IPV. These changes remained elevated six months later, which suggests long-term changes in attitudes that may have clinical implications for their work.

The educational intervention was delivered to healthcare providers who were currently part-time students, taking further education in mental healthcare. A Cochrane review on training healthcare providers to respond to IPV against women concluded that training programs can be successfully conducted at universities, targeting various healthcare providers [6]. The intervention aimed to enhance healthcare providers' knowledge of MR-IPV by integrating theoretical concepts with practical field experiences. Moreover, the intervention was organized as a seminar day, as part of their educational curriculum, which demonstrated the relevance and applicability of the topic to their ongoing studies. This organization and the content of the lectures may explain why the educational intervention had such a strong impact on how the healthcare providers perceived the relevance of the topic.

Various pedagogical tools were selected for this educational intervention to enhance the learning experience. The main component was the expert presentations, which provided healthcare providers with accurate and up-to-date knowledge and insights from experienced professionals in the field. Sharing ideas and perspectives through reflection exercises and group activities enabled healthcare providers to learn from one another and connect theoretical concepts to real-life scenarios, facilitating knowledge acquisition, critical thinking, and practical skill development. These methods align with findings from a Cochrane review on training healthcare providers to respond to IPV, highlighting the integration of diverse pedagogical approaches in intervention programs [6]. However, the review also found that training programs on IPV vary in duration, content, and dynamics, emphasizing the need for authors to describe their intervention in sufficient detail to allow for replication [32]. Very few intervention studies on IPV have provided comprehensive details about the training program [6]. Therefore, we have included a detailed description of this educational intervention to ensure transparency and allow for replication.

This study is one of the few studies that have specifically focused on the legal obligations and practical application of MR-IPV training for healthcare providers, making it an important contribution to the field. Training healthcare providers has been proposed to be a cost-effective and cost-saving intervention from a societal perspective [33], supporting further implementation of training programs on MR-IPV. The World Health Organization recently highlighted that violence prevention interventions that have been proven effective in pilot studies can be expanded, such as by replicating them in another geographic location or expanding their coverage to wider areas [2]. In this context, we would like to emphasize that replicating or scaling up this educational intervention in new settings would require adaptation – both to region-specific needs and values, and to the specific legal context, given the large differences in the legal contexts of MR-IPV worldwide. The skills healthcare providers need to respond to disclosures of IPV and fulfill their MR-IPV obligations will depend on the legal context.

### Strengths and limitations

This study has some limitations that should be considered when interpreting the findings. One limitation is the relatively small sample size, which may reduce the generalizability of the results and increase the risk of Type II errors. Additionally, the study relied on self-report measures, which may be subject to bias. However, the use of a validated instrument enhances the validity of the results. Social desirability bias could also have influenced the results, particularly at T1, as participants may have expressed more favorable attitudes toward MR-IPV after the intervention to align with perceived expectations from the educators or peers, rather than reflecting their genuine beliefs. While this bias could theoretically have affected findings related to changes in attitudes, we do not believe it would have biased the results showing increased knowledge about MR-IPV.

The participants included in this study may differ from the broader population of healthcare providers, both demographically and behaviorally. Including healthcare providers who were taking further education in mental healthcare might have introduced a selection bias toward those with a specific interest in the topic. Their pursuit of further education may make them more motivated to increase their knowledge and open to changing their attitudes, compared to the broader population. Moreover, the study was conducted in Norway, where specific legal frameworks, cultural norms, and healthcare systems can shape how healthcare providers approach IPV and MR. While the intervention shows promising results for this group, future research should explore the impact of the intervention among a broader range of healthcare providers, in settings beyond Norway, and over an extended period of time.

In addition, we did not repeatedly measure the participants' professional experiences with MR and IPV, based on feedback from participants in the pilot study. However, upon reflection, we realize that collecting repeated measures of their experiences could have deepened the understanding of our findings. This is particularly relevant for understanding their professional experiences with MR. For example, we found that only ten participants believed prior to the intervention that they had ever worked on cases where MR was required, even though half of the participants had worked on cases involving *severe* IPV. Since very few participants reported knowing the MR law and its criteria prior to the intervention, it is possible that their perceptions of previous experiences would have changed after increasing their knowledge about MR-IPV. After the intervention, all participants agreed that severe IPV should always be reported to the police, which starkly contrasts with their reported practice of MR. This finding is consistent with previous research that has shown that few professionals have reported IPV under MR [9], and that reporting rates can be low despite professionals’ extensive experience with IPV [34]. Furthermore, we note that half of the participants reported encountering IPV victims and/or perpetrators in the 12 months prior to the intervention. This suggests that many of them continued to experience IPV cases after the intervention, which may have influenced their responses at the six-month follow-up. To improve future studies, it may be worth considering collecting repeated measures on participant experiences for a more comprehensive analysis.

## Conclusions

In this study, we developed an educational intervention on MR-IPV that covered several related topics, with a strong emphasis on the legal context and barriers to reporting. The intervention included various pedagogical tools that facilitated knowledge acquisition, critical thinking, and practical skill development. The educational intervention had a positive impact on the healthcare providers' knowledge and several attitudes. Our main findings were that the intervention significantly increased healthcare providers' knowledge about MR laws, guidelines, and criteria relevant to assessing whether MR-IPV applies in clinical contexts. After the intervention, most healthcare providers considered MR to be a useful instrument and generally complied with MR requirements. While set in a Norwegian context, the intervention along with its findings can have relevance to a broader global context of healthcare responses to IPV.

The healthcare providers participating in this study had worked on average 8.5 years in their respective fields, where most of them had encountered victims and/or perpetrators of IPV during their career. Despite these experiences, only 3% reported prior to the intervention knowing the MR law and few participants were well-informed about the guidelines and application criteria for MR-IPV. Moreover, only ten participants meant prior to the intervention that they had ever worked on cases where MR was required, although half of the healthcare providers had worked on cases with *severe* IPV. These findings demonstrate the need for continuous learning and evidence-based training programs to equip healthcare providers with essential knowledge and skills.

## Supplementary Information

Supplementary Material 1.

## Data Availability

The datasets analysed during the current study are available from the corresponding author on reasonable request.

## Abbreviations

IPV: Intimate Partner Violence
MR: Mandatory reporting
MR-IPV: Mandatory Reporting of Intimate Partner Violence
RD: Risk Difference
CI: Confidence Intervals
