# Supplementary material for: An educational intervention study on mandatory reporting of intimate partner violence: changes in knowledge and attitudes among healthcare providers
Source: BMC Med Educ. 2024 Oct 11;24:1124. doi: 10.1186/s12909-024-06120-8 (PMC11468273; doi:10.1186/s12909-024-06120-8)
Supplement: Supplementary file 1 — Supplementary Material 1. [file 12909_2024_6120_MOESM1_ESM.docx]

**Supplementary file 1: Excerpts from the Questionnaire**

***Section D) Knowledge and attitudes towards mandatory reporting and client confidentiality***

| Knowledge of mandatory reporting | | | |
| --- | --- | --- | --- |
|  | Yes | To some extent | No |
| Do you know the mandatory reporting law? | 1 | 2 | 3 |
| Do you know the mandatory reporting law within your field? | 1 | 2 | 3 |

| What is closest to your perception of “mandatory reporting”? | | | | |
| --- | --- | --- | --- | --- |
|  | Furthest from my perception | | Closest to my perception | |
| Severe intimate partner violence should always be reported to the police. | 1 | 2 | 3 | 4 |
| I am the one who decides whether I will use mandatory reporting | 1 | 2 | 3 | 4 |
| Client confidentiality takes precedence over mandatory reporting | 1 | 2 | 3 | 4 |
| Mandatory reporting takes precedence over client confidentiality | 1 | 2 | 3 | 4 |

| The mandatory reporting law refers to a range of criminal offenses (laws), including abuse in close relationships | | | | | |
| --- | --- | --- | --- | --- | --- |
|  | Completely  disagree | | Completely  agree | | Does not exist in my field |
| Do you believe the mandatory reporting law considers the central factors in the risk situation | 1 | 2 | 3 | 4 | 9 |
| Has knowledge of mandatory reporting changed the way you work? | 1 | 2 | 3 | 4 | 9 |
| Do you believe mandatory reporting is a useful tool? | 1 | 2 | 3 | 4 | 9 |

| To what extent do you agree with the following statements? | | | | | |
| --- | --- | --- | --- | --- | --- |
|  | Completely  disagree | Partly  disagree | Partly agree | Completely agree | Don’t’ know |
| Everyone has the right to a physician/therapist/healthcare provider/contact person with absolute client confidentiality | 1 | 2 | 3 | 4 | 9 |
| If I disclose confidential information, patients/clients/consumers will lose trust in me, regardless of justification | 1 | 2 | 3 | 4 | 9 |
| If I disclose confidential information, I will feel unprofessional, regardless of justification | 1 | 2 | 3 | 4 | 9 |
| The patient's wish matters most; if they don't want to report to the police, I won't do it | 1 | 2 | 3 | 4 | 9 |
| I find other ways to cooperate with, for example, the police, without using mandatory reporting | 1 | 2 | 3 | 4 | 9 |
| Unless the patient poses a significant risk to others, the help services should not overrule the wishes and decisions of adults | 1 | 2 | 3 | 4 | 9 |
| Mandatory reporting is important to ensure that victims of intimate partner violence do not have to report the risk of severe abuse and/or of intimate partner homicide themselves | 1 | 2 | 3 | 4 | 9 |
| I generally comply with the mandatory reporting requirements when treating patients/clients/consumers | 1 | 2 | 3 | 4 | 9 |
| The distinction between the Health Personnel Act and mandatory reporting is unclear | 1 | 2 | 3 | 4 | 9 |
| I find that my professional autonomy is reduced due to mandatory reporting | 1 | 2 | 3 | 4 | 9 |

| Triggering mandatory reporting | | | |
| --- | --- | --- | --- |
|  | No | To some extent | Yes |
| Are you informed about the criteria that should be used to make a decision regarding the application of mandatory reporting within your field? | 1 | 2 | 3 |

***Section E) Knowledge and attitudes towards guidelines and risk assessment***

| To what extent do you agree with the following statements? | | | | | |
| --- | --- | --- | --- | --- | --- |
|  | Completely  disagree | Partly  disagree | Partly agree | Completely agree | Don’t’ know |
| I am well-informed about the current guidelines in my field | 1 | 2 | 3 | 4 | 9 |
| I often disagree with the recommendations in the guidelines | 1 | 2 | 3 | 4 | 9 |
| The guidelines are integrated into the way I work | 1 | 2 | 3 | 4 | 9 |
| Guidelines are often not easily accessible | 1 | 2 | 3 | 4 | 9 |
| I miss a comprehensive record of guidelines | 1 | 2 | 3 | 4 | 9 |
| I have confidence in the guidelines published by my professional union | 1 | 2 | 3 | 4 | 9 |
